# Obesity and elevated blood pressure among adolescents in Lagos, Nigeria: a cross-sectional study

**DOI:** 10.1186/1471-2458-12-616

**Published:** 2012-08-07

**Authors:** Abiola A Oduwole, Taiwo A Ladapo, Iretiola B Fajolu, Ekanem N Ekure, Olufunmilayo F Adeniyi

**Affiliations:** 1 Department of Paediatrics, College of Medicine, University of Lagos, Lagos, Nigeria; 2 Department of Paediatrics, Lagos University Teaching Hospital, Lagos, Nigeria

**Keywords:** Adolescents, Blood pressure, Body mass index, Obesity, Overweight

## Abstract

**Background:**

Childhood obesity and associated hypertension are major public health concerns globally. This study aimed to determine the prevalence of obesity and the associated risk of high blood pressure among Nigerian adolescents.

**Methods:**

A cross-sectional school-based study of 885 apparently healthy adolescents was performed. Weight, height and blood pressure (BP) were measured using standard methods. Body mass index (BMI) was calculated and categorized by age, sex and percentile. Obesity and overweight were defined as: ≥ 95th and 85th to < 95th percentiles, respectively, for age, sex and height. Subjects were sub-categorized into age 10–13 years (A) and 14–17 years (B). The odds ratio for pre-hypertensive and hypertensive range BP by age and BMI were generated. Significance was set at P < 0.05.

**Results:**

The prevalence of overweight and obesity were 13.8% and 9.4%, respectively. The prevalence of hypertensive range systolic BP in obese versus normal BMI females was 16% versus 23% (p=0.00) and 12.1% versus 6.4% (p=0.27) in males. The prevalence of hypertensive range diastolic BP in obese versus normal BMI females was 12% versus 1.4% (p=0.00) and 15.2% versus 3.5% (p=0.01) in males. BMI in group B was significantly associated with pre-hypertensive and hypertensive range systolic BP in overweight (P = 0.01, P = 0.002) and obese subjects (P = 0.00, P = 0.00) and with hypertensive range diastolic BP (P = 0.00) only in obese subjects. The only significant association in group A was between obesity and pre-hypertensive range diastolic BP (P = 0.00).

**Conclusion:**

The prevalence of hypertensive range BP among obese Nigerian adolescents was high. Screening for childhood obesity and hypertension, and long-term follow-up of obese adolescents into adulthood are recommended.

## Background

Childhood obesity is increasingly being recognized worldwide as a major public health problem reaching epidemic proportions [1,2]. According to the World Health Organization, an estimated 40 million children under 5 years of age worldwide are overweight [2]. Initially thought to be a disease of the Western world, this disease is now increasing in the developing parts of the world [2-4]. In Nigeria, the prevalence rates obtained from previous studies ranged from 0.3% to 5% [5-8], while in the United States of America, prevalence is reportedly as high as 13–14% [9]. The United States Preventive Services Task Force (USPSTF) has therefore recommended that clinicians screen all children aged 6 years and above for obesity and apply appropriate interventions [9].

The public health significance of childhood obesity (defined as age- and gender-specific body mass index (BMI) greater than the 95th percentile on the BMI growth chart) [9] stems from its association with other comorbidities such as insulin resistance, hypertension, type 2 diabetes, hyperlipidaemia, liver and renal disease, reproductive dysfunction and certain cancers [2,10]. Hypertension has been demonstrated in several populations to have a positive correlation with obesity and overweight, with obese children having a 3-fold increased risk of developing hypertension compared with their non-obese counterparts [3,11-14]. This is believed to be partly responsible for the rising incidence of childhood hypertension worldwide. Persistent childhood hypertension is often predictive of future adult hypertension, cardiovascular disease and early death [15-17]. The true burden of obesity-related hypertension in Nigeria is unknown as data are scarce. Therefore, the objective of this study was to determine the prevalence of obesity and overweight among a group of otherwise healthy Nigerian adolescents, and the association between their BMI and blood pressure (BP).

## Methods

### Study area and population

This was a cross-sectional study carried out at a secondary school in an urban area of the city of Lagos in Lagos State, Nigeria. The State has an estimated population of 17 million out of a national estimate of 150 million. With an urban population of about 85%, Lagos is the largest urban conglomeration in Nigeria. The population of the urban districts of Lagos consists of low to high income socioeconomic classes, thus any selected district is representative of the general urban population.

The school was selected by a simple random method. The State is divided into six educational districts from which one was randomly selected using a table of random numbers from a list provided by the state school board. A list of schools in the district was also obtained and this was stratified into single and mixed gender schools. The study centre was randomly selected using a table of random numbers from the list of coeducational schools in the selected educational district zone. All pupils of the selected school who were in a state of apparent good health were eligible for the study. Exclusion criteria were a previous diagnosis of an underlying kidney or heart disease, known hypertension, acute illness at the time of the study and failure of students or their parents/guardian to give consent. Formal approval for the study was obtained from the school authorities and informed consents were obtained from both the participating students and their parents. Ethical approval was obtained from the ethics and research committee of the Lagos University Teaching Hospital.

### Data collection

The study team first visited the school during a general assembly, where the nature and importance of the study were explained to the students. The apparatus to be used, a standard mercury sphygmomanometer and digital weighing scale, was demonstrated, and a brief explanation of the procedures was given. Consent forms were distributed to the pupils through the school authorities and these were collected over a 2-week period prior to commencement of the study. Subsequently all eligible participants were enrolled into the study. Demographic data, including name, age, and sex were obtained from the class registers and entered into a database.

### Measurements

All measurements were taken by the investigators who had received adequate training in these procedures. Weight was measured with a digital weighing scale after checking for zero error at each measurement and the reading was taken to the nearest 0.1 kg. Subjects were weighed barefoot, standing still and without support, in light clothing. Belts and other accessories were removed and pockets emptied. Height was measured with a stadiometer to the nearest 0.5 cm with the subjects barefoot, standing erect with heels together and looking straight ahead. The lower edge of the socket was in the same horizontal plane as the external auditory meatus, with heels and back against the height rule [18]. Weight and height were used to determine BMI. Pupils were categorized by age and sex using the US Centers for Disease Control and Prevention (CDC) BMI growth charts as follows: obese: ≥95th percentile; overweight: 85th to <95th percentile; normal: 5th to <85th percentile; underweight: < 5th percentile [19].

BP was measured using a standard mercury sphygmomanometer. Subjects were seated comfortably with legs uncrossed and feet resting on a firm surface. BP cuffs with bladder width at least 40% of the arm circumference at a point mid-way between the olecranon and the acromion and length covering 80–100% of the arm circumference were used. During deflation of the cuff, systolic BP (SBP) and diastolic BP (DBP) were recorded at the 1st and 5th Korotkoff sounds, respectively, with readings taken to the nearest 2 mmHg. Two BP readings were obtained, with a 5-minute interval between readings, and the mean of the readings was taken as the subjects’ BP [20,21]. BP readings were classified according to the recommendations of the National Blood Pressure Education Programme: Pre-hypertension ≥90th to <95th percentile; hypertension ≥95th percentile [21].

### Statistical analysis

Data were analysed using the SPSS version 17 and EPI info version 3.53 software. BMI and percentiles were generated using the Nutstat component of the Epi Info software [22] which uses 2000 CDC growth charts for boys and girls. Values were expressed as means (± standard deviation) for age, weight, height, BMI and BP. Pearson correlation statistics were used to determine correlation coefficients between BMI and various variables. The odds ratios of elevated BP in the pre-hypertensive and hypertensive ranges by age and BMI category were determined. A P-value < 0.05 was considered significant.

## Results

Nine hundred and eleven pupils met the eligibility criteria and were recruited into the study and 885 with complete data were subsequently analysed. There were 400 males and 485 females giving a male:female ratio of 1:1.21, and the age range was 9–18 years (mean, 13.04 ± 1.92). Mean age, BMI, and DBP of both sexes were similar (P = 0.98, 0.10, 0.23, respectively), while their mean weights, heights and SBP were significantly different (P < 0.05), the females being heavier and the males taller. There were 209 (23.2%) pupils with BMI above the 85th percentile for age and sex, 124 of whom were overweight and 85 were obese, giving prevalence rates of 13.8% and 9.4%, respectively. It was observed that the playground that was available during school break time and after school hours was generally poorly utilized by the students.

The BMI category by sex is displayed in Table 1. It reflects a higher prevalence of both overweight and obesity in females than males. Pearson's correlation analysis between BMI and various parameters in all the study subjects demonstrated a positive correlation between BMI and weight, height, SBP and DBP in males: (r = 0.59, P < 0.01), (r = 0.26, P < 0.01), (r = 0.31, P < 0.01), and (r = 0.27, P < 0.01), respectively, and in females: (r = 0.1, P < 0.01 ), (r = 0.54, P < 0.01), (r = 0.1, P < 0.01) and (r = 0.27, P < 0.01), respectively. None of the underweight children had elevated BP in the hypertensive range.

**Table 1:** BMI category by sex

	**BIM**				
**Sex**	**Underweight N (%)**	**Normal N (%)**	**Overweight N (%)**	**Obese N (%)**	**Total N (%)**
Male	15 (1.7)	311 (35.1)	41 (4.6)	33 (3.7)	400 (45.2)
Female	8 (0.9)	346 (39.1)	81 (9.2)	50 (5.7)	485 (54.8)
Total	23 (2.6)	657 (74.2)	122 (13.8)	83 (9.4)	885 (100)

Percentages of the total number of subjects are in brackets. BMI: Body mass index.

### BMI and BP

In Table 2, BP was stratified by BMI and gender. The overall prevalence of pre-hypertensive and hypertensive range SBP was 5% and 6%, respectively, and for DBP was 12.5% and 4%, respectively. A steady increase in the prevalence of both hypertensive and pre-hypertensive range SBP and DBP with increasing BMI category was demonstrated. The prevalence of hypertensive range SBP was significantly higher in obese females compared with their counterparts with normal BMI: 16% *versus* 2.3% (P < 0.05), while in males it was 12.1% *versus* 6.4% (P > 0.05). The prevalence of hypertensive range DBP in the obese compared with those with normal BMI was 15.2% *versus* 3.5% (P < 0.05) in males and 12% *versus* 1.4% (P < 0.05) in females. Similarly, the prevalence of both SBP and DBP in the pre-hypertensive range was higher in obese pupils compared with their counterparts with normal BMI but the difference was only significant for DBP in both sexes (P < 0.05).

**Table 2:** Blood pressure categories stratified by BMI and sex

	**SBP**			**DBP**		
	**NT (%)**	**PHTN (%)**	**HTN (%)**	**NT (%)**	**PHTN (%)**	**HTN (%)**
Underweight						
Male	14 (93.3)	1 (6.7)	0 (0.0)	12 (80.0)	3 (20.0)	0 (0.0)
Female	8 (100)	0 (0.0)	0 (0.0)	7 (87.5)	1 (12.5)	0 (0.0)
Normal						
Male	282 (90.7)	9 (2.9)	20 (6.4)	266 (85.5)	34 (10.9)	11 (3.5)
Female	322 (93.1)	16 (4.6)	8 (2.3)	304 (87.9)	37 (10.7)	5 (1.4)
Overweight						
Male	34 (82.9)	4 (9.8)	3 (7.3)	33 (80.5)	6 (14.6)	2 (4.9)
Female	66 (81.5)	6 (7.4)	9 (11.1)	65 (80.2)	10 (12.3)	6 (7.4)
Obese						
Male	26 (78.8)	3 (9.1)	4 (12.1)	20 (60.6)	8 (24.2)	5 (15.2)
Female	37 (74.0)	5 (10.0)	8 (16.0)	32 (64.0)	12 (24.0)	6 (12.0)

NT: Normotension; PHTN: Pre-hypertension; HTN: Hypertension.

**Figure 1:**
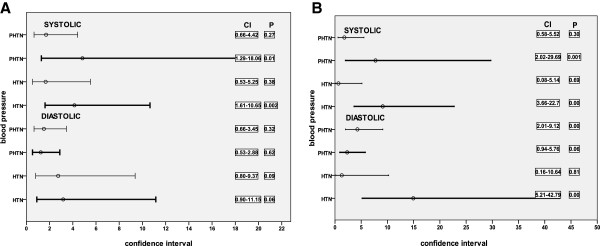
**Odds ratio of hypertension by age group in overweight (A) and obese (B) subjects. **Values on the right represent the P values and 95% confidence intervals. Abscissas represent odds ratios and 95% confidence intervals. Lighter line: 10–13 years; darker line: 14–17 years.

Subjects were grouped into two age categories: A (10–13 years) and B (14–17 years). One subject aged 9 years and six aged 18 years and above were excluded from the new groups because of their small numbers. There were 246 males and 291 females in group A and 154 males and 194 females in group B. The odds ratio for hypertensive range SBP and DBP for each age group by BMI category are shown in Figure 1A and B. In group A, only DBP in the pre-hypertensive range was significantly associated with BMI (P < 0.05) and this was in obese subjects. However, in group B, BMI was significantly associated with pre-hypertensive and hypertensive range SBP in the overweight (P < 0.05) and obese groups (P < 0.05). It was also similarly associated with hypertensive range DBP in the obese group (P < 0.05).

## Discussion

The high prevalence of overweight and obesity observed in this study is alarming especially when compared with the prevalence in a small number of previous reports from comparable locations, which ranged from 0.3% to 5% [5-7]. An exception to this however was the study by Owa [23], also in the same environment which reported a prevalence of 18%. The wide variation in prevalence is largely attributable to differences in population type, geographical location and age range studied [5-7]. In the study by Owa, where the prevalence of obesity was similar to that of the current study, the age range studied was wide, consisting of 5–15 year olds, and there was no distinction between overweight and obese subjects. The urban location of our study may also partly explain the relatively high prevalence, as an urban preponderance of childhood obesity has been well described in the developing world [3,7,24].

In a study among adolescents performed in the same environment as this study about a decade ago [7], a low prevalence of obesity of 0.4% was reported. The authors noted, as have other African authors [3,5,23], that with increasing affluence in our society associated with a gradual adoption of Western lifestyles [3,24,25] and reduction in physical activity, childhood obesity may become a potential public health problem in Nigeria and other developing countries. This is especially true of highly industrialized cities like Lagos, which may account for the much higher prevalence in the current study that is similar to those obtained from studies in the Western world [11,14]. The low level of physical activity in our cohort, despite the availability of the playground, is worthy of note. Urbanization and acquisition of a Western lifestyle may also apply to the subjects in the study by Owa [23], who were from the elite class and lived in a highly developed part of the area of study. This is in contrast to the developed world where obesity seems to be more predominant among rural dwellers [11,26].

In our study, BP increased with age, as has been documented by other studies [3,15,24,27]. In addition, SBP and DBP were positively correlated with BMI in both sexes. In the total study population, there were 7- and 2-fold increases, respectively, in the risk of hypertensive range SBP in obese females and males compared with their normal BMI counterparts. The risk of hypertensive range DBP was about 4-fold and almost 2-fold for males and females, respectively, compared with normal BMI subjects. In addition, the overall risk for pre-hypertensive range BP in obese subjects was 2–2.5 times higher than for normal BMI subjects. The association between BMI and BP has been attributed to various interacting complex systems that have been observed in obese children. They include over-activity of the renin-angiotensin and sympathetic nervous systems, insulin resistance and abnormalities in vascular structure and function [28].

A higher risk of hypertensive range SBP and DBP was documented amongst the cohort in group B (the older age group) implying that if weight control interventions are implemented from early childhood, obesity and its attendant comorbidities may be prevented. The USPSTF recommendation for obesity screening from the age of 6 years [9] is therefore appropriate, as obesity-related hypertension has been demonstrated even in pre-adolescent children [29,30].

The increase in prevalence of hypertensive range SBP in obese children compared with their counterparts with normal BMI was significant in females and not in males. An increase in hypertensive range SBP with age in children with normal BMI was observed only in males in our cohort. This phenomenon has been attributed to hormone-related sexual dimorphism [27,30]. Even though we did not apply the Tanner staging of puberty, the non-observance of this phenomenon amongst females implies that obesity may exert a greater influence over SBP than hormonal-related pubertal changes. Further studies will be needed to verify this.

Publications on the relationship between BMI and BP are limited in our environment. From the few accessible, the general trend was an increase in BP with BMI, though with some variations [27,31]. In a study that specifically correlated obesity with hypertension in adolescents [27], the authors demonstrated 2- and 3-fold increases in point hypertension in overweight/obese males and females, respectively, compared with their normal BMI contemporaries in an urban area. In the semi-urban area, the increase was about 5-fold for girls but was absent for boys. However, the study did not distinguish between overweight and obese subjects and SBP and DBP. It is possible that if overweight and obesity were separately assessed, a more significant increase in BP with BMI would be observed in obese subjects.

The findings in this current study are strikingly similar to that of a Canadian study [11] that reported a 19.5% prevalence of hypertension in obese children compared with 4% in non-obese children. Similarly, the prevalence of pre-hypertension was 18.2% and 5.7% in obese and non-obese children, respectively. In the same study, there was a higher prevalence of obesity-related hypertension in children aged 13–17 years compared with the lower age group of 4–12 years. These findings were independent of family history of hypertension or kidney disease, similar to our observations and further substantiated by a similar recent study from Sudan [32].

A recently concluded longitudinal follow-up of 13-year-old adolescents into young adulthood in the USA [16] revealed that high BP and BMI act independently and have an additive effect in predicting the highest levels of cardiovascular risk in young adults. It can therefore be inferred that an increase in the prevalence of adolescent hypertension may herald an increase in the prevalence of adult hypertension, cardiovascular morbidity, and mortality in Nigeria. The threat this poses on the already inundated health-care system is enormous and calls for action by all stakeholders to combat this epidemic at every level of care.

A limitation of this study was that BP readings were obtained on a single day and hence the persistence of elevated BP could not be ascertained. This is significant as some studies have demonstrated a reduction in the prevalence of elevated BP following repeated measurements in the same cohort [33,34]. In a large Swiss study [33], there was a 5-fold decrease in the proportion of children with elevated BP following triplicate BP measurements. However, overweight or obesity still accounted for 37% of cases of hypertension in their cohort. In view of this limitation, a follow-up to this study with recall of all the students with pre-hypertensive and hypertensive range BP is planned. Information about sexual maturation and dietary intake such as level of salt consumption which may affect BP was also not available.

## Conclusion

The prevalence of overweight and obesity in our study in Lagos was high. Obesity significantly increased the risk of both systolic and diastolic BP in the hypertensive range especially in older adolescents. We recommend that screening for childhood obesity and its comorbidities, of which hypertension is prominent, should be intensified at all levels of care, and affected children should be referred for appropriate intervention. The role of health education and mass public enlightenment on the dangers of some aspects of Westernization of the lifestyle, such as childhood obesity and its comorbidities, cannot be over-emphasized. Follow-up studies to determine the long-term outcome of childhood obesity should be initiated.

## Abbreviations

USPSTF: United States Preventive Services Task Force; BMI: Body Mass Index; CDC: US Centers for Disease Control; SBP: Systolic blood pressure; DBP: Diastolic blood pressure; PHTN: Pre-hypertension; HTN: Hypertension.

## Competing interests

The authors hereby declare that there were no competing interests.

## Authors’ contributions

OAO and LTA: Conceptualization of the study, study design, proposal writing, data collection, data analysis, discussion and editing of the final draft for publication. FIB: Study design, proposal writing, data collection, data analysis, discussion and editing of the final draft for publication. EEN: Conceptualization of the study, study design and editing of the final draft for publication. AOF: Proposal writing, study design and data collection. All authors approved the final draft of this article prior to submission.

## Authors’ information

A O, the lead author of this article is the current President of the African Society for Paediatric and Adolescent Endocrinology. This work was done as part of her commitment to advancing the frontiers of medical research in child and adolescent endocrinology especially in the African community. Her advocacy role in this position will also be strengthened by the availability of such data.

## Pre-publication history

The pre-publication history for this paper can be accessed here:

http://www.biomedcentral.com/1471-2458/12/616/prepub
